# Assessment of maternal dietary patterns and their relationship with c-reactive protein in pregnancy

**DOI:** 10.1093/eurpub/ckac130.102

**Published:** 2022-10-25

**Authors:** P van der Pligt, SJ McNaughton, K Kuswara, G Abbott, S Islam, S Ebrahimi, S Ellery

**Affiliations:** 1 Institute for Physical Activity and Nutrition, Deakin University, Geelong, Australia; 2 School of Exercise and Nutrition Sciences, Deakin University, Burwood, Australia; 3 The Ritchie Centre, Hudson Institute of Medical Research, Clayton, Australia; 4 Department of Obstetrics and Gynaecology, Monash University, Clayton, Australia

## Abstract

**Background:**

Inflammation during pregnancy including elevated C-reactive protein (CRP) is associated with adverse pregnancy outcomes. Understanding relationships between CRP and modifiable factors such as dietary patterns is key to identifying opportunities for pregnancy intervention. This study assessed change in adherence to the Dietary Approaches to Stop Hypertension (DASH) and Mediterranean diet (MED-diet) from early to late-pregnancy and the relationship between adherence to both dietary patterns at early-pregnancy with plasma CRP at early and late-pregnancy.

**Methods:**

Women (n = 215) attending antenatal clinics at Monash Health, Melbourne were recruited at 10-20 weeks gestation. Medical history and blood samples were collected at 5 antenatal visits. Adapted DASH and MED-diet scores were calculated from Food Frequency Questionnaires completed at early ([mean±SD] 15±3 weeks) and late (36±1 week) pregnancy. CRP was measured in maternal plasma samples collected at early and late-pregnancy. Adjusted linear regression assessed associations of early-pregnancy DASH and MED-diet scores with early and late-pregnancy plasma CRP.

**Results:**

DASH score at early (23.5±4.8) and late (23.5±5.2) pregnancy was not significantly different (p = 0.971). There was no statistically significant change in MED-diet score from early (3.99±1.6) to late-pregnancy (4.08±1.8) (p = 0.408), however, MED-diet adherence and plasma CRP at early pregnancy were significantly and inversely associated (β= -0.14 [95%CI= -0.27, -0.01], p = 0.039).

**Conclusions:**

Adherence to the MED-diet in early pregnancy may be beneficial in reducing inflammatory markers and assisting optimal pregnancy outcomes. Assessment of dietary patterns is important to assist identifying modifiable factors which impact maternal and child health.

**Key messages:**

- Adherence to the MED-diet during pregnancy may be important in reducing inflammation.
- Assessment of maternal dietary patterns can assist informing early pregnancy intervention.

